# Serotypes and Antimicrobial Resistance of Human Nontyphoidal Isolates of *Salmonella enterica* from Crete, Greece

**DOI:** 10.1155/2014/256181

**Published:** 2014-04-22

**Authors:** Sofia Maraki, Ioannis S. Papadakis

**Affiliations:** Department of Clinical Microbiology, Parasitology, Zoonoses and Geographical Medicine, University Hospital of Heraklion, Heraklion, 71 110 Crete, Greece

## Abstract

We report on the serotype distribution and the antimicrobial resistance patterns to 20 different antimicrobials of 150 *Salmonella enterica* strains isolated from stools of diarrhoeal patients on the island of Crete over the period January 2011-December 2012. Among the *S. enterica* serotypes recovered, Enteritidis was the most prevalent (37.3%), followed by Typhimurium (28.7%) and Newport (8.7%). No resistance was detected to extended-spectrum cephalosporins and carbapenems. Rates of resistance to ampicillin, amoxicillin/clavulanic acid, chloramphenicol, tetracycline, and cotrimoxazole were 9.3%, 4%, 2%, 15.3%, and 8.7%, respectively. Resistance to ≥4 antibiotics was primarily observed for serotypes Typhimurium and Hadar. Enteritidis remains the predominant serotype in Crete. Although low resistance to most antimicrobials was detected, continued surveillance of susceptibility is needed due to the risk of resistance.

## 1. Introduction

Salmonellosis is one of the most common and widely distributed foodborne diseases, causing approximately 93.8 million illnesses and 155,000 deaths annually around the world [1]. In the European Union (EU),* Salmonella *causes approximately 6.2 million human cases each year and represents a considerable economic impact costing €3 billion a year [2]. With around 23,000 hospitalizations and almost 400 deaths per year,* Salmonella *species were estimated to cause the most annual deaths among foodborne pathogens in the United States [3]. Although most* Salmonella* infections result in mild to moderate self-limiting gastroenteritis requiring little or no intervention, serious extraintestinal complications, such as septicemia, endocarditis, meningitis, and osteomyelitis, may occur [4]. Antimicrobial treatment is essential in cases of invasive disease, at the extremities of age, and in the presence of underlying disease or immunosuppression [4]. The global emergence of multiple drug resistance among* Salmonella enterica* strains is a serious public health concern because treatment may fail if the infecting strain is resistant to the prescribed agent [5]. It has been shown that outbreaks caused by antimicrobial-resistant, nontyphoidal salmonellae were associated with an increased rate of hospitalization compared with outbreaks caused by pansusceptible salmonellae [6]. Moreover, studies conducted in Canada and Denmark found excess death rates associated with resistant* Salmonella *infection [7, 8].

In the current report, we present the serotype distribution and antimicrobial resistance patterns among* S. enterica* strains isolated from patients with acute gastroenteritis isolated at our institution between 2011 and 2012.

## 2. Materials and Methods

### 2.1. Study Design and Data Collection

We studied* S. enterica* strains isolated from fecal samples of patients with gastroenteritis cared for at the University Hospital of Heraklion, Crete, Greece, between January 2011 and December 2012, that is, over a 24-month period. Duplicate samples were excluded and only a single isolate from epidemiologically related outbreaks was included. The laboratory participated in the WHO Global-Salm-Surv, a network involved in* Salmonella *surveillance. In addition to the serotype and the antibiotic susceptibility patterns, the name, sex, age, and date of culturing were recorded in an Access database (Microsoft Corporation, Seattle, WA).

### 2.2. Bacteriology

Isolates were biochemically identified using conventional microbiological methods, the API 20E system, and the automated Vitek 2 system (both products of BioMérieux, Marcy l'Etoile, France) [9]. Serotyping was performed using commercial antisera (Statens Serum Institut, Denmark) and the Kauffmann-White scheme [9]. Quality control of serotyping and antibiotic susceptibility testing was performed annually through the WHO. Antimicrobial susceptibility was performed for 20 antibiotics either by the disk diffusion method or the automated Vitek 2 system for ampicillin, amoxicillin/clavulanic acid, piperacillin, piperacillin/tazobactam, ticarcillin, ticarcillin/clavulanic acid, imipenem, meropenem, aztreonam, cefotaxime, ceftriaxone, ceftazidime, cefepime, chloramphenicol, nalidixic acid, ciprofloxacin, norfloxacin, tetracycline, cotrimoxazole, and nitrofurantoin. The minimal inhibitory concentration (MIC) of ciprofloxacin was determined using the agar dilution method in accordance with CLSI guidelines for* Salmonella* strains resistant to nalidixic acid [10]. Decreased ciprofloxacin susceptibility was defined as an MIC of ≥0.125 *μ*g/mL [11].* Escherichia coli* ATCC 25922 was used as a reference standard. Intermediately resistant strains were included in the resistant category. Multidrug resistance was defined as resistance to ≥4 antibiotics.

## 3. Results

During a 2-year period, 150 strains of* S. enterica* were isolated from faecal samples of patients having diarrhoeal illness. Eighty-two of them (54.7%) were obtained from male patients and sixty-eight (45.3%) from females. Of those, 124 (83%) were Greeks, while 17% were foreigners. Among the foreigners, 45% were tourists, mainly from Central and Northern Europe, while 55% were aliens, economic immigrants. Sixty-seven percent of the samples were obtained at the emergency room or at outpatient departments, while 33% were taken from hospitalized patients. Patient age ranged from 1 month to 87 years (median 8 years). More than one-third, 36.7%, of the patients were children below 6 years and 21.3% were between 6 and 15 years of age (Table 1).

Additionally, nontyphoidal salmonellae were isolated from the blood cultures of 3 patients with bacteraemia and from the synovial fluid of a patient with arthritis.

*Salmonella* isolates belonged to thirty serotypes with Enteritidis being the most frequent (37.3%), followed by Typhimurium (28.7%). Serotype distribution by study year is shown in Table 2.

Table 3 shows antimicrobial resistance to different antimicrobial agents. Rates of resistance to ampicillin, amoxicillin/clavulanic acid, ticarcillin, piperacillin, chloramphenicol, tetracycline, cotrimoxazole, nalidixic acid, ciprofloxacin, and norfloxacin were 9.3%, 4%, 10%, 10%, 2%, 15.3%, 8.7%, 13.3%, 1.3%, and 1.3%, respectively. Among antibiotics not shown in Table 3, the following overall rates of resistance were identified: piperacillin/tazobactam 2% and ticarcillin/clavulanic acid 5.3%.

For 7 antibiotics, the four extended-spectrum cephalosporins (cefotaxime, ceftriaxone, ceftazidime, and cefepime), the two carbapenems (imipenem and meropenem), and the monobactam, aztreonam, no resistant isolates were identified.

The highest level of resistance among all tested antibiotics was seen for nitrofurantoin (overall resistance 48%) ranging from 20% for Hadar to 92.3% for Newport isolates. Resistance to ≥4 antibiotics was seen in 4.7% of the isolates. Multidrug resistance was almost exclusively limited in two serotypes, that is, Hadar (60%) and Typhimurium (9.3%).

Resistance to ciprofloxacin was identified in two strains, while decreased susceptibility was found in twelve strains belonging to serotype Enteritidis (MIC = 0.125 *μ*g/mL, four strains; MIC = 0.25 *μ*g/mL, eight strains), four to serotype Hadar (MIC = 0.125 *μ*g/mL, three strains; MIC = 0.25 *μ*g/mL, one strain), one to serotype Heidelberg (MIC = 0.25 *μ*g/mL), and one to serotype Newport (MIC = 0.5 *μ*g/mL).

**Figure 1:**
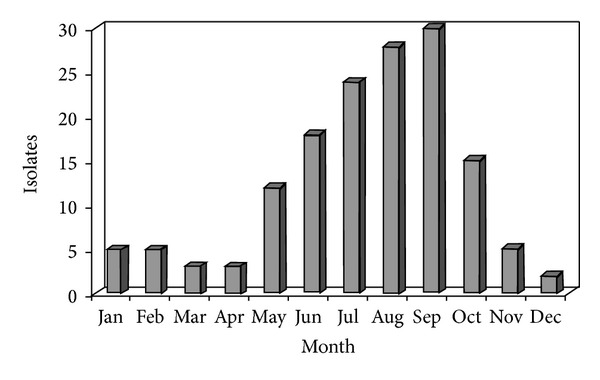
Monthly incidence of* Salmonella* enteritis infection.

*S. enterica* enteritis occurred throughout the year with substantially higher incidence in summer (Figure 1).

## 4. Discussion and Conclusion

Nontyphoidal* Salmonella* species are important foodborne pathogens worldwide accounting for a large number of outbreaks and sporadic cases [1]. In recent years, a decreasing trend in the incidence of salmonellosis has been observed in Crete and in all over Greece as well [12, 13]. The decrease observed from 1995 to 2012 has been particularly sharp (63.1%) for* Salmonella enterica* serotype Enteritidis [12]. There has also been a statistically significant reduction in reported cases rates across the EU between 2006 and 2010, which resulted from the decreased* Salmonella *infections caused by* S. enterica* serotype Enteritidis [13, 14]. This is most likely due to the implementation of control measures against* Salmonella* within the poultry industry (e.g., vaccination of laying hens and broilers), improved hygiene, and education of food workers [13, 14]. Despite this decline Enteritidis is still the predominant serotype in Greece and in the other European countries [13].

Isolation rates were highest in children aged 0–5 years. This finding regarding the young age of infection is consistent with other studies which have shown that younger children are at greater risk of infection [15, 16]. Additionally, children with diarrhea—being at risk of dehydration—are more likely to see a doctor and therefore to have a stool examination. There is a huge detection bias in the age distribution of culture confirmed cases. Increased exposure to antibiotics, which predispose to* Salmonella* infection through suppression of normal gut flora and the decreased gastric acidity, might contribute to the difference in illness threshold [16]. There is a clear seasonal trend for* Salmonella* infections with rates increasing during the summer months, reaching a peak in August-September. It has been shown that a higher ambient temperature leads to an increase of salmonellosis notifications, possibly through an increase in the reproduction of salmonellas at various points of the food chain [17].

The overall low rate of resistance to ampicillin (9.3%) in our study is similar to that reported previously but much lower than that described by us for similar gastrointestinal isolates implicated in diarrhoeal disease in the University Hospital of Heraklion during the period 1995–1999 (31.5% versus 9.3%) [11, 12]. Rates of resistance to ampicillin varied according to serotype. All isolates belonging to serotype Enteritidis were susceptible to ampicillin, while resistance was mainly detected among isolates of serotypes Hadar and Typhimurium with 60% and 20.9% being resistant, respectively. Results published by Enternet, corresponding to data of 10 European countries, reported for 2004 significantly higher rates of resistance to ampicillin for serotype Typhimurium than for Enteritidis (57% versus 7%) [18].

Regarding the third-generation cephalosporins, no resistant isolates were identified. In Crete, a single* S. enterica* serotype Virchow strain producing TEM-52 extended-spectrum *β*-lactamase (ESBL) was detected in 2002 [19]. ESBL-producing* Salmonella enterica* serotypes Typhimurium, Brandenburg, Blockley, and Hadar have been isolated in Greece since 1994 [20]. In Europe the percentage of nontyphoidal* Salmonella* isolates resistant to third-generation cephalosporins is low, while in the USA an increase in resistance from 0.2% to 3.4% has been detected from 1996 to 2009 [18, 21]. Recently, 2 strains producing carbapenemase, one KPC-2 and one NDM, have been detected in the USA [22, 23]. The emergence of such strains resistant to all beta-lactam antibiotics available is of great concern because of the limitation of therapeutic choices for patients with invasive* Salmonella* infections.

Over the study period resistance to fluoroquinolones tested was seen in 1.3% of the isolates, while 12% exhibited reduced susceptibility. Of note is the significant increase in nalidixic acid resistance from 2.7% in 2000–2004 to 13.3% in 2011-2012 [11]. The global increase in the prevalence of fluoroquinolone resistance or reduced susceptibility in* Salmonella *species constitutes a major concern, since these pathogens have been associated with clinical failures of therapy and with a significant burden of hospitalization [24]. A 5-year survey of antimicrobial resistance in 134,310 human nontyphoidal* Salmonella* isolates from 10 European countries showed that the overall resistance to nalidixic acid increased from 14% to 20% and in serovar Enteritidis from 10% to 26% [18]. Rates of full resistance to ciprofloxacin increased from 0.2% to 0.9% [18]. In the USA, the percentage of nontyphoidal* Salmonella* isolates resistant to ciprofloxacin increased from 0.4% to 2.4%, from 1996 to 2009 [21].

Of note is an increase in cotrimoxazole resistance from 2.7% in 2000–2004 to 8.7% in 2011-2012 (*P* = 0.004) [11]. This percentage is also increased compared with that previously reported for childhood gastrointestinal* Salmonella* isolates in the University Hospital of Heraklion during the period 1993–2010 (5.5% versus 8.7%) [25]. The same trend was also reported from the Greek National Reference Center for* Salmonella* and* Shigella*, in an 8-year survey of antimicrobial resistance in 1,548 nontyphoidal* Salmonella *isolates. An increase in resistance to cotrimoxazole from 5.7% in 1990 to 9.6% in 1997 has been found [26].

Regarding nitrofurantoin, high resistance rates (48%) were detected. Studies in Portugal and the UK showed a remarkable incidence of* Salmonella *isolates from several sources (predominantly human and poultry) resistant to nitrofurantoin. This is likely due to the veterinary use of nitrofurans in food animal production units [27].

Multiresistance was uncommon (4.7%) and limited to two* S. enterica* serotypes, that is, Hadar and Typhimurium. The multidrug-resistant (MDR)* Salmonella* Typhimurium definitive phage type (DT) 104 with chromosomally encoded resistance to ampicillin, chloramphenicol, streptomycin/spectinomycin, sulfonamides, and tetracyclines (ACSSpSuT) was first identified in the early 1990s and subsequently caused numerous outbreaks worldwide [28]. Recently, there has been a decline in the incidence of pentaresistant DT104 Typhimurium in several European countries [18] and an international increase of the monophasic serovar 4,[5],12:i:- isolates expressing resistance to ampicillin, streptomycin, sulphonamides, and tetracyclines [29]. In Greece, this monophasic serovar ranks third among all serovars in humans and fourth in EU 2010 [13, 30].

In conclusion, the present study revealed low antibiotic resistance among gastrointestinal* S. enterica* strains isolated during the years 2011-2012 in the island of Crete. However, continuing local and national surveillance of the development of antimicrobial resistance is necessary. Local surveillance data are important for the clinicians who need guidance with empirical therapy, and information at national level is essential in order to implement appropriate control measures.

## Figures and Tables

**Table 1:** Age distribution of the patients with *S. enterica* enteritis.

Age group (yr)	Number (%) of isolates
0–5	55 (36.7)
6–15	32 (21.3)
16–45	27 (18)
46–60	9 (6)
≥61	27 (18)

**Table 2:** Serotype distribution of 150 *S. enterica* isolates over the study years.

Year	Enteritidis	Typhimurium	Newport	Hadar	Litchfield	Thompson	Muenchen	Others^a^	Total
2011	32	20	8	4	2	1	2	18	84
2012	24	23	7	1	1	2	0	8	66

Total	56	43	15	5	3	3	2	26	150

^a^Others. Serotypes: Agona, Blockley, Braenderup, Breukelen, Bloomsbury, Bovismorbificans, Choleraesuis,   Infantis,   Inpraw, Kedougou, Kentucky, Lindenburg, Manchester, Manhattan, Muenster, Heidelberg, Oakey, Saintpaul, Senftenberg, Seremban, Stanleyville, Tennyson, Tshiongwe (1 isolate each).

**Table 3:** Antibiotic resistance patterns of different *S. enterica* serotypes isolated from stools over a 2-year period.

Serotype	AMP	AMX/CLA	TIC	PIP	CTX	CRO	NAL	CIP	NFX	CMP	TET	TMP-SMX	NTR	Resistant to ≥4 abx*
S. Enteritidis	0^a^	0	0	0	0	0	17.8	0	0	0	0	1.8	60.7	0
	0/56^b^	0/56	0/56	0/56	0/56	0/56	10/56	0/56	0/56	0/56	0/56	1/56	34/56	0/56

S. Typhimurium	20.9	7	20.9	20.9	0	0	0	0	0	7	30.2	23.3	34.9	9.3
	9/43	3/43	9/43	9/43	0/43	0/43	0/43	0/43	0/43	3/43	13/43	10/43	15/43	4/43

S. Newport	0	0	0	0	0	0	7.7	0	0	0	7.7	0	92.3	0
	0/13	0/13	0/13	0/13	0/13	0/13	1/13	0/13	0/13	0/31	1/13	0/31	12/13	0/13

S. Hadar	60	40	60	60	0	0	80	0	0	0	60	0	20	60
	3/5	2/5	3/5	3/5	0/5	0/5	4/5	0/5	0/5	0/5	3/5	0/5	1/5	3/5

Others	6.1	3	9.1	9.1	0	0	15.1	6.1	6.1	0	18.2	6.1	30.3	0
	2/33	1/33	3/33	3/33	0/33	0/33	5/33	2/33	2/33	0/33	6/33	2/33	10/33	0/33

Total	9.3	4	10	10	0	0	13.3	1.3	1.3	2	15.3	8.7	48	4.7
	14/150	6/150	15/150	15/150	0/631	0/631	20/150	2/150	2/150	3/150	23/150	13/150	72/150	7/150

*abx: antibiotics.

AMP: ampicillin; AMX/CLA: amoxicillin-clavulanic acid; TIC: ticarcillin; PIP: piperacillin; CTX: cefotaxime; CRO: ceftriaxone; NAL: nalidixic acid; CIP: ciprofloxacin; NFX: norfloxacin; CMP: chloramphenicol; TET: tetracycline; TMP-SMX: cotrimoxazole; NTR: nitrofurantoin.

^a^% (percentage of resistant isolates/serotype).

^b^Number of resistant isolates/total number of isolates in serotype.
